# Beyond unseen entities: Auditable cold-start evaluation for drug interaction prediction

**DOI:** 10.1371/journal.pcbi.1014927

**Published:** 2026-11-10

**Authors:** Xiaoqing Ru, Yansu Wang, Quan Zou, Mengting Niu

**Affiliations:** 1 The Quzhou Affiliated Hospital of Wenzhou Medical University, Quzhou People’s Hospital, Quzhou, China; 2 Institute of Fundamental and Frontier Sciences, University of Electronic Science and Technology of China, Chengdu, China; 3 Department of Integrative Biotechnology, College of Biotechnology and Bioengineering, Sungkyunkwan University, Suwon, Republic of Korea; 4 School of Electronic and Communication Engineering, Shenzhen Polytechnic University, Shenzhen, China; Guangxi University, CHINA

## Abstract

Reliable generalization is essential for deploying drug–drug interaction (DDI) and drug–target interaction (DTI) models in drug discovery, pharmacovigilance, and clinical decision support. However, strong performance on random splits or weakly constrained evaluations may not reflect how well a model generalizes to new drugs, targets, or deployment conditions. In pairwise prediction, test instances may still resemble training examples in molecular structure, sequence, or network context. Such overlap can inflate performance estimates and make interpolation appear to be extrapolation. In this Perspective, we argue that cold-start evaluation should be treated as an auditable deployment claim. We organize common evaluation settings by their source and scope of novelty, ranging from random splits and entity-disjoint protocols to structure-level, temporal, domain-disjoint, and many-domain evaluations. The L0–L5 framework clarifies which deployment claim each setting is designed to test. Coverage, overlap, and distance quantify the resulting train–test discrepancy. We further provide a practical checklist for leakage control, validation alignment, candidate-pair construction, and domain-level risk reporting. This Perspective clarifies the boundaries and evidential scope of cold-start evaluation, providing a common basis for interpreting and comparing results across DDI and DTI studies.

## 1. Introduction

Evaluation design is central to assessing generalization in DDI and DTI prediction. Different evaluation protocols impose different forms of train–test separation, which can substantially affect reported performance and the conclusions drawn from benchmark results [1–7]. In DDI prediction, Celebi and colleagues [8] showed that drug-wise and pairwise disjoint cross-validation produced lower performance estimates than conventional cross-validation, highlighting the effect of evaluation design on apparent model performance. In DTI prediction, Bai and colleagues [9] similarly found that random splitting produced overly optimistic estimates compared with more restrictive splitting strategies, including cold-drug, scaffold, and hierarchical-clustering splits. These findings show that benchmark performance should be interpreted in relation to the novelty and train–test separation imposed by the evaluation protocol. This issue becomes particularly important in cold-start evaluation, where different protocols may represent substantially different forms of novelty.

Recent studies show that nominally unseen test entities may still remain close to the development distribution [10,11]. In DTA prediction, Zhang and colleagues [4] found that conventional test sets were dominated by samples highly similar to the training data, with model performance declining markedly as similarity decreased. In emerging DDI prediction, Shen and colleagues [2] noted that commonly used known/new drug splits are often constructed in an i.i.d. manner and therefore do not explicitly model distribution changes between the two drug sets. These studies show that unseen entity identity alone fails to enforce the information boundary assumed at deployment.

Cold-start novelty can arise from different sources, including new drugs in distinct chemical subspaces, new targets from different families or mechanistic regimes, changing assay platforms, and evolving database versions [12–15]. Different sources of novelty represent different generalization challenges and may induce shifts of different severity. These dimensions should therefore be evaluated separately when interpreting cold-start results. Fig 1 illustrates the underlying issue: a pair can be nominally new while remaining connected to development data through shared entities and the surrounding relational structure.

**Fig 1:**
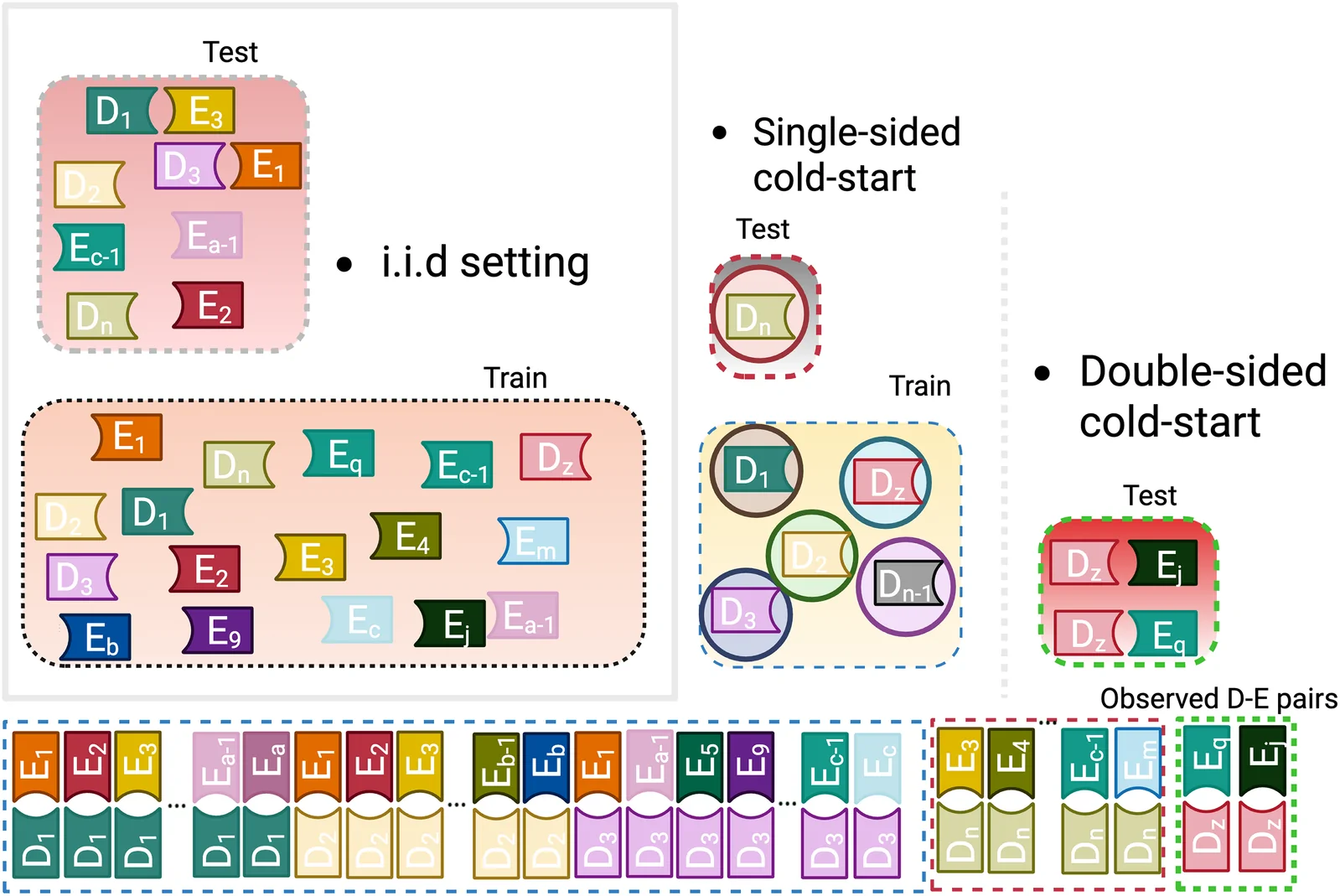
Random, single-sided, and double-sided cold-start settings for pairwise prediction. D and E denote the interacting entities; both are drugs in DDI, whereas in DTI they correspond to a drug and a target. Random splitting does not enforce entity disjointness; single-sided and double-sided cold start withhold one and both entities, respectively, from model development. Created in BioRender. Wang, Y. (2026) https://BioRender.com/934ry5s.

We therefore view cold-start evaluation in DDI and DTI prediction as an auditable deployment claim. Its interpretation requires clarity about what is new, what information remains available during model development, and how large the resulting shift is. The L0–L5 framework developed below organizes common evaluation protocols around these questions and provides the basis for the subsequent auditing and reporting recommendations.

## 2. Why current cold-start terminology is insufficient

The term cold start is used inconsistently across DDI and DTI studies [16–18]. Dewulf and colleagues [1] distinguished DDI tasks involving an unknown drug–drug pair, one unknown drug, and two unknown drugs as different cold-start settings. In the Therapeutics Data Commons [19], cold splitting is instead defined by specifying the entity type to be withheld, such as the drug in DTI, and can be extended to multiple entity types. These usages differ in both the held-out unit and the prediction task, making the label “cold start” insufficient to identify the evaluation condition on its own. This ambiguity also complicates comparison across studies. Cold-start protocols should be accompanied by an operational description of what is withheld and any additional structural, temporal, or domain constraints.

## 3. From unseen entities to auditable deployment claims

For a cold-start result to support a deployment claim, the evaluation protocol should define the intended discrepancy between model development and testing [20–23]. We characterize this discrepancy through three auditable questions:

- Q1. Source of novelty. What is expected to be new at deployment: entity identity, chemical structure or protein sequence, evidence source or database version, deployment environment, or data provenance?
- Q2. Information-boundary protection. Which cross-boundary information is controlled during model development, including shared entities, close structural analogues or sequence homologues, overlapping network neighborhoods, and label-correlated metadata unavailable in the intended deployment setting [24]?
- Q3. Shift severity**.** How large is the train–test separation, and how is it quantified, for example through cross-boundary structural- or sequence-similarity distributions, neighborhood-overlap rates, inter-domain distances, or temporal gaps?

The answers to these questions define the evidential scope of a cold-start result and the generalization claim that the evaluation can support.

### 3.1 A deployment-aligned framework for evaluation protocols

To operationalize the preceding questions, we organize commonly used DDI and DTI protocols into six evaluation settings (Fig 2). L0–L5 describe the source and scope of novelty represented by an evaluation, but the settings are not mutually exclusive: entity, structural, temporal, and domain constraints may be combined within the same protocol. Evaluations can be described by the applicable settings together with the specific constraints imposed. The framework is intended to clarify the evaluation condition and the generalization question being assessed, rather than to define a fixed hierarchy of difficulty.

**Fig 2:**
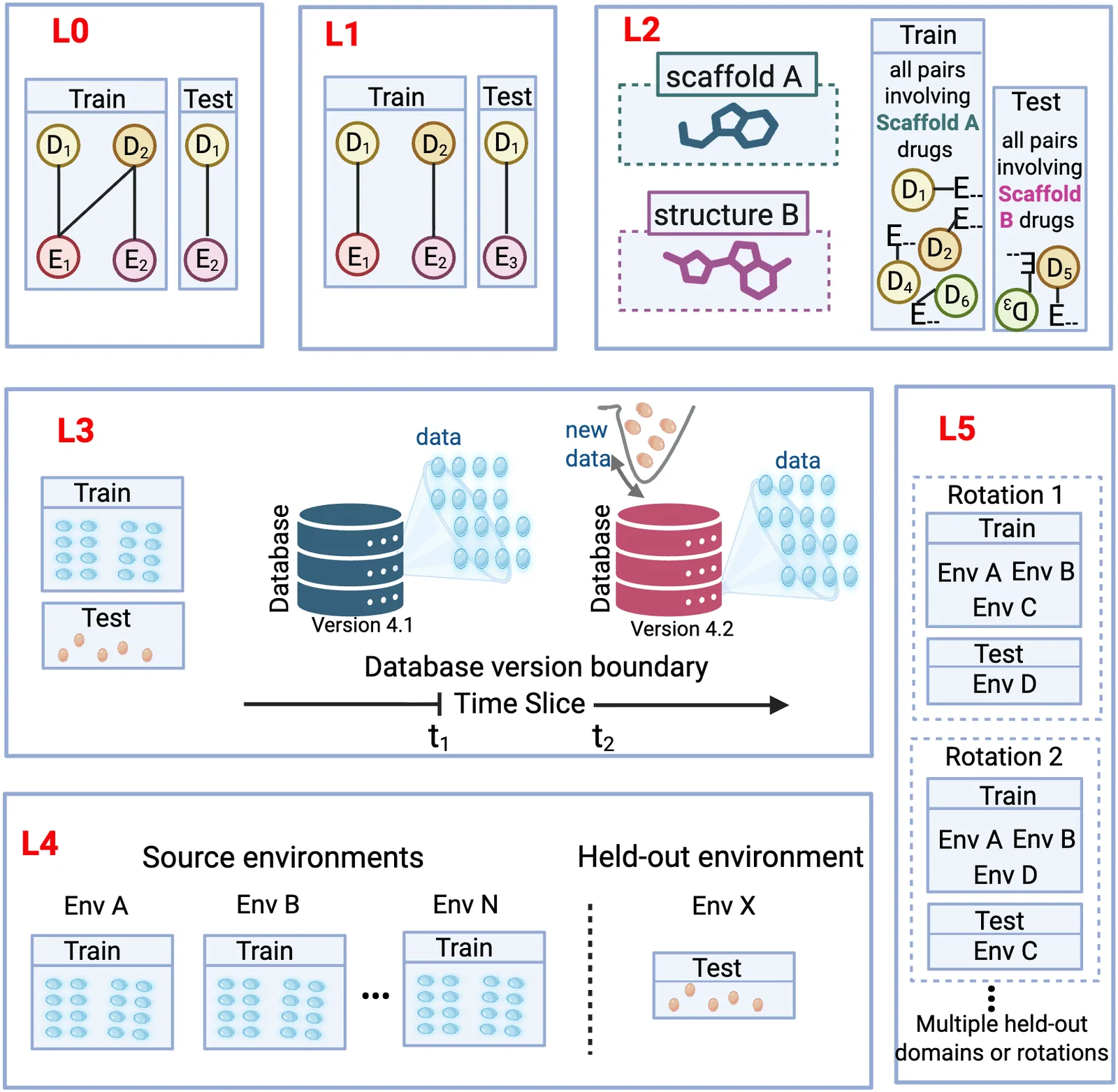
Deployment-aligned framework for cold-start evaluation protocols in DDI and DTI prediction. L0–L5 organize evaluation settings by the source and scope of novelty, and multiple constraints may be combined within the same protocol. Created in BioRender. Wang, Y. (2026) https://BioRender.com/33z4d7i.

#### i. L0 Random split.

L0 uses random partitioning and serves as an interpolation baseline. Because test instances may remain close to training instances in chemical, sequence, or network space, L0 assesses performance near the observed data distribution and does not support cold-start or out-of-distribution generalization claims [25–27].

#### ii. L1 Entity-disjoint protocols.

Under L1, each test pair contains either one entity unseen during model development (single-sided cold start) or two unseen entities (double-sided cold start) [16,18,28]. It tests generalization to unseen entities within a development space that may still contain close structural analogues, sequence homologues or overlapping relational neighborhoods. L1 supports entity-level novelty claims, but not necessarily structure-level or domain-level extrapolation.

#### iii. L2 Structure-level extrapolation.

L2 imposes separation in chemical or sequence space, typically through scaffold splits, similarity thresholds or similarity-based clustering [29–31]. It tests whether a model can generalize beyond local chemical or sequence neighborhoods and may be combined with L1, for example by pairing a scaffold-disjoint drug split with cold-target evaluation [32].

#### iv. L3 Prospective evaluation by time or database version.

L3 uses temporal cutoffs or database-version boundaries to approximate prospective evaluation under evolving evidence conditions. Later data may differ in entity composition, chemical or sequence space, and evidence-generation or curation practices, with consequences for predictive performance, uncertainty, and calibration. Temporal and version-based splits may therefore combine multiple sources of distribution shift, and reports should specify the cutoff or version boundary and quantify the resulting train–test discrepancy [33–35].

#### v. L4 Domain-disjoint evaluation.

L4 evaluates generalization to a deployment-relevant domain excluded from model development [36–38]. Here, a domain is a prespecified group of samples that share a common data-generating or deployment condition, such as an assay platform, data source, experimental setting, or another grouping with clear deployment meaning [39]. Similarity-based criteria may also contribute to domain construction. Accordingly, split construction alone does not distinguish L2 from L4. The distinction lies in the role assigned to the resulting groups: L2 uses similarity to enforce separation in chemical or sequence space, whereas L4 treats the groups as deployment-relevant domains.

#### vi. L5 Many-domain stress testing.

L5 extends L4 from a single held-out domain to multiple held-out domains or repeated domain rotations, testing whether model performance remains stable across heterogeneous deployment conditions [40,41].

Performance should be reported for individual domains and summarized across domains. Macro-averaging gives equal weight to each domain, whereas sample-size-weighted averaging weights domains according to their sample sizes. When domain sizes are heterogeneous, the two summaries provide complementary views of performance. Aggregate performance should also be accompanied by worst-domain or lower-quantile performance, domain-level uncertainty intervals, and calibration across domains or shift-severity strata [42]. Tail-sensitive summaries should be interpreted cautiously when domains are small or few. Many-domain formulations in healthcare provide a useful conceptual template, although direct DDI/DTI benchmarks of this type remain limited.

### 3.2 Separating evaluation setting from shift severity

The L0–L5 framework describes the source and scope of novelty represented by an evaluation, while shift severity reflects the degree of separation between development and test data. A stringent scaffold split may induce greater train–test separation than a short-interval temporal split, and evaluations within the same domain-disjoint setting may also differ substantially in shift severity [43,44]. Studies should therefore report the applicable L0–L5 setting or combination of settings together with quantitative measures of the resulting train–test discrepancy [45]. An evaluation label should not be treated as a proxy for the magnitude of distribution shift.

## 4. Auditing the induced train–test discrepancy

Once an evaluation protocol is specified, the resulting train–test discrepancy should be assessed independently of model performance. We characterize this discrepancy along three complementary dimensions: coverage, overlap, and distance. Coverage reflects the extent to which test samples fall within the support of the development data. It can be quantified using nearest-neighbor structural or sequence similarity, or by the proportion of test samples meeting a predefined support criterion [46,47]. Overlap captures information shared across the train–test boundary, such as repeated entities or common graph neighbors [48]. Distance uses prespecified, task-relevant covariates or representations to quantify distribution-level separation between development and test data [49–52]. Reports should specify the representation, metric, and aggregation used for each measure.

For L5, these measures should be computed for each held-out domain or domain rotation and reported alongside domain-specific performance. Joint reporting can distinguish broad performance degradation with increasing shift severity from failures concentrated in particular domains. Table 1 summarizes the minimum reporting requirements for the evaluation protocol, induced discrepancy, and performance claims.

**Table 1:** Minimum reporting recommendations for deployment-aligned evaluation of DDI and DTI prediction under controlled distribution shifts.

No.	Reporting item	Minimum information to report	Failure mode addressed
1	Task definition and deployment-time information	Define the prediction outcome and unit of inference; specify the entity- and pair-level information available and unavailable at deployment.	Ambiguous task or deployment assumptions; unsupported generalization claims.
2	Evaluation setting(s) and deployment claim	Identify the applicable L0–L5 setting(s), combined constraints, deployment condition, and the generalization claim.	Incomparable evaluation conditions; overinterpretation of an L0–L5 designation.
3	Split construction and partition logic	Specify held-out units, endpoint constraints, similarity criteria, temporal or version boundaries, domain-assignment rules, and treatment of cross-boundary pairs.	Irreproducible partitions; leakage or selection bias.
4	Candidate pair universe and label semantics	Define pair eligibility and filtering; treatment of duplicate, reverse, and self-pairs; positive, negative, and unlabeled pairs; negative-sampling and class prevalence.	Incomparable pair construction, label semantics, or sampling.
5	Domain or cluster definition and scale	Report defining variables and assignment rules, deployment interpretation, group counts and sizes, and handling of small groups.	Weakly justified group definitions; unstable domain-level or tail-risk estimates.
6	Audit of the induced train–test discrepancy	Report coverage, overlap, and distance with the representation, metric, and aggregation used; provide endpoint-specific summaries when relevant.	Generalization claims unsupported by the achieved separation or shift severity.
7	Development boundary and leakage control	State the information available at each stage of model development and exclude test data from data-dependent decisions; disclose potential leakage from external resources, pretrained representations, graph features, or temporal information.	Optimistic estimates caused by direct or indirect test-information leakage.
8	Validation alignment	Explain how validation approximates the test constraints without accessing the test partition; justify any mismatch	Model selection misaligned with the intended evaluation condition.
9	Performance distribution and risk-aware reporting	For multiple domains or rotations, report domain-specific performance, macro- and sample-size-weighted summaries, uncertainty, and tail-sensitive measures; stratify by shift severity when relevant.	Aggregate results masking poor or unstable performance in particular domains.
10	Reproducibility materials	Provide **entity lists, candidate-pair universes**, split identifiers, group assignments, rotation schedules, data versions, seeds, preprocessing specifications, and reconstruction code or configuration; document restrictions and deterministic alternatives.	Evaluations that cannot be reconstructed, audited, or compared.

**Applicability note.** Items 1–4, 7, 8, and 10 apply across evaluation settings. Item 5 applies to domain- or cluster-based protocols, Item 6 to evaluations beyond interpolation and is particularly important for L4–L5, and Item 9 to multi-domain or repeated-rotation evaluations.

## 5. Conclusions and outlook

The central message of this Perspective is that the interpretation of cold-start performance depends on both the evaluation condition and the train–test discrepancy. The L0–L5 framework and discrepancy audits make these two aspects explicit, providing a clearer basis for cross-study comparison and appropriately scoped generalization claims.

Current evaluation practice should make the development–test boundary explicit and auditable. Studies should document split construction and data provenance, align validation with the intended test condition, and prevent test information from entering model development. For prospective and many-domain evaluations, uncertainty and calibration should be evaluated alongside predictive performance. Many-domain studies should additionally report domain-specific results rather than aggregate metrics alone.

Looking ahead, two questions deserve particular attention. One is how entity, structural, temporal, and domain shifts interact when they occur simultaneously. The other is how model performance changes as shift severity increases and whether distinct failure regimes emerge. Answering these questions will help define the boundaries of reliable generalization under realistic and evolving deployment conditions.
